# A QTL for conformation of back and croup influences lateral gait quality in Icelandic horses

**DOI:** 10.1186/s12864-021-07454-z

**Published:** 2021-04-14

**Authors:** Maria K. Rosengren, Heiðrún Sigurðardóttir, Susanne Eriksson, Rakan Naboulsi, Ahmad Jouni, Miguel Novoa-Bravo, Elsa Albertsdóttir, Þorvaldur Kristjánsson, Marie Rhodin, Åsa Viklund, Brandon D. Velie, Juan J. Negro, Marina Solé, Gabriella Lindgren

**Affiliations:** 1 Department of Animal Breeding and Genetics, Swedish University of Agricultural Sciences, Uppsala, Sweden; 2 The Agricultural University of Iceland, Borgarnes, Iceland; 3 Genética Animal de Colombia Ltda, Bogotá, Colombia; 4 The Icelandic Agricultural Advisory Centre, Reykjavík, Iceland; 5 Department of Anatomy, Physiology and Biochemistry, Swedish University of Agricultural Sciences, Uppsala, Sweden; 6 School of Life & Environmental Sciences, University of Sydney, Sydney, Australia; 7 Department of Evolutionary Ecology, Doñana Biological Station, CSIC, Seville, Spain; 8 Livestock Genetics, Department of Biosystems, KU Leuven, Leuven, Belgium

**Keywords:** Back, Backline, Conformation, Croup, High-density genome scan, Icelandic horse, Lateral gait quality, Novel QTL

## Abstract

**Background:**

The back plays a vital role in horse locomotion, where the spine functions as a spring during the stride cycle. A complex interaction between the spine and the muscles of the back contribute to locomotion soundness, gait ability, and performance of riding and racehorses. Conformation is commonly used to select horses for breeding and performance in multiple horse breeds, where the back and croup conformation plays a significant role. The conformation of back and croup plays an important role on riding ability in Icelandic horses. However, the genes behind this trait are still unknown. Therefore, the aim of this study was to identify genomic regions associated with conformation of back and croup in Icelandic horses and to investigate their effects on riding ability. One hundred seventy-seven assessed Icelandic horses were included in the study. A genome-wide association analysis was performed using the 670 K+ Axiom Equine Genotyping Array, and the effects of different haplotypes in the top associated region were estimated for riding ability and additional conformation traits assessed during breeding field tests.

**Results:**

A suggestive quantitative trait loci (QTL) for the score of back and croup was detected on *Equus caballus* (ECA) 22 (*p*-value = 2.67 × 10^− 7^). Haplotype analysis revealed two opposite haplotypes, which resulted in higher and lower scores of the back and croup, respectively (*p*-value < 0.001). Horses with the favorable haplotype were more inclined to have a well-balanced backline with an uphill conformation and had, on average, higher scores for the lateral gaits tölt (*p*-value = 0.02) and pace (*p*-value = 0.004). This genomic region harbors three genes: *C20orf85*, *ANKRD60* and *LOC100056167. ANKRD60* is associated with body height in humans*. C20orf85* and *ANKRD60* are potentially linked to adolescent idiopathic scoliosis in humans.

**Conclusions:**

Our results show that the detected QTL for conformation of back and croup is of importance for quality of lateral gaits in Icelandic horses. These findings could result in a genetic test to aid in the selection of breeding horses, thus they are of major interest for horse breeders. The results may also offer a gateway to comparative functional genomics by potentially linking both motor laterality and back inclination in horses with scoliosis in humans.

**Supplementary Information:**

The online version contains supplementary material available at 10.1186/s12864-021-07454-z.

## Background

Associations of body measurements with locomotor health and sports performance have been reported in many different breeds, including Icelandic horses [1–11]. Discriminant analyses have shown that several morphological features distinguish with high accuracy between low-class and high-class Icelandic horses with respect to different riding ability traits [3]. The most important features for gait ability in Icelandic horses are the height of the horse at front compared to hind (uphill conformation) with well-balanced backline, croup proportions and width of chest [1, 3]. The analyses also indicated the disadvantage of a forward inclination in the back or a sway back [3]. Conformation of the back and croup thus play a major role on riding ability in Icelandic horses.

The Icelandic horse official breeding goal promotes five-gaited horses with a functional and aesthetically pleasing conformation [12]. Zoometric measurements and subjective scores for conformation and riding ability traits are recorded at breeding field tests. Genetic correlations between conformation of back and croup, and gait qualities have been estimated as moderate to high (0.19–0.54) [1]. Furthermore, moderate heritabilities (0.29–0.31) have been estimated for the subjectively scored back and croup trait [1, 13] and the objectively measured zoometric traits pertaining to conformation of back and croup (0.20–0.25) [3]. For the subjectively scored riding ability traits, the heritability estimates range from 0.18 (walk) to 0.60 (pace) [1, 13].

Despite conformation traits being moderately heritable in the Icelandic horse, only mutations in the Myostatin gene have previously been associated with conformation traits, i.e. estimated breeding values of neck, withers and shoulders [14]. In other horse breeds, as well as other species, many different genes have been shown to influence body size. *LCORL*, *NCAPG* and *HMGA2* are major genes known to regulate body size in mammals including humans, cattle, sheep, dogs and horses [15–23]. These genes, along with other genes such as *ZFAT* and *LASP1*, affect not only the body size of the horse but more specifically the height at withers [15, 24, 25]. Three novel missense variants located in the *ADAMTS17*, *OSTN* and *GH1* genes explained 61% of the variance of withers height in Shetland pony-related breeds [26]. Other additional quantitative trait loci have also shown significant associations with morphometric angular measurements, with regions on chromosomes ECA28 and ECA29 associated with poll angle in horses [27]. However, the genes behind many other conformation traits are still unknown.

Considering the heritability of conformation of back and croup and its genetic correlation with riding ability, we hypothesized that major genetic factors of importance for back and croup also influence gait quality in Icelandic horses. Therefore, the aim of this study was to identify genomic regions associated with conformation of back and croup in Icelandic horses and investigate their effects on riding ability traits assessed at breeding field tests.

## Results

### Genome-wide association analysis for conformation of back and croup

**Fig. 1:**
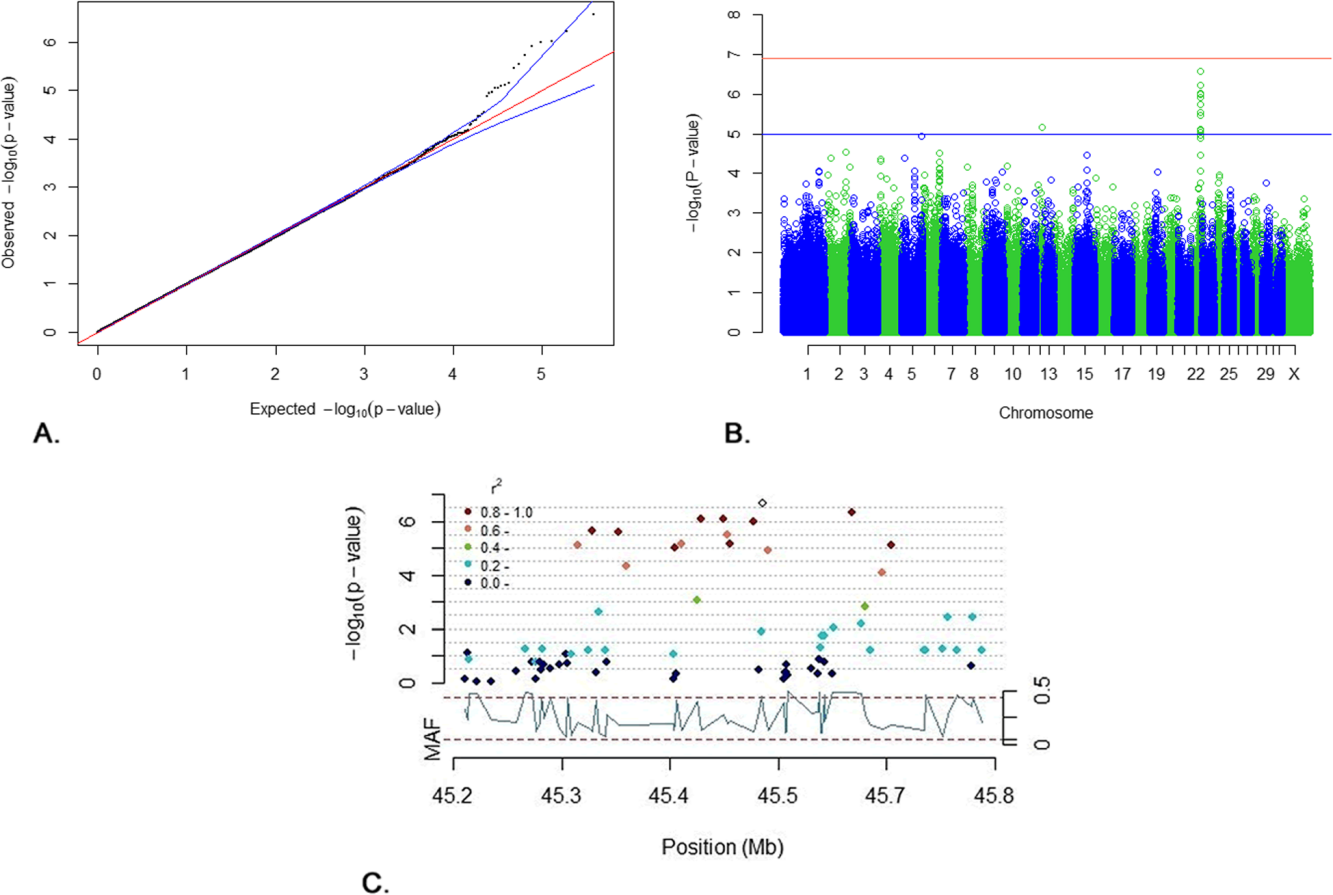
GWA results for the score of back and croup. **a.** QQ plot where the blue lines represent the 0.05–0.95 confidence interval. The estimated lambda value was 0.98 (se 2.55 × 10^− 5^). **b.** Manhattan plot from the mixed model association analysis. The red horizontal line indicates Bonferroni significance threshold (*p* < 6.9 × 10^− 8^) and the blue horizontal line indicates the suggestive genome-wide significance level (*p* < 1.0 × 10^− 5^). **c**. LD Manhattan plot on ECA22 with the top SNP as an open circle. Thirteen SNPs reached the suggestive threshold of which ten were in LD. All positions refer to EquCab3.0

In total, 383,896 SNPs (373,041 autosomal and 10,855 X chromosomal) and 177 horses passed QC and were included in the GWA analysis. Thirteen SNPs located on ECA22: 45347522–45,662,708 reached the suggestive threshold (*p* < 1.0 × 10–5) of which ten were in LD (r2 ≥ 0.8) (Fig. 1). Additionally, one single SNP reached the suggestive threshold on ECA12 (Fig. 1). A summary of the GWA results for the 50 top SNPs is presented in Additional file 1.

### Haplotype analysis

The haplotype analysis revealed two opposite haplotypes which resulted in higher and lower scores for back and croup (*p*-value < 0.001) (Table 1). Thirty-four horses were homozygous for the haplotype associated with a higher score and 28 horses homozygous for the haplotype associated with a lower score of back and croup. Five different haplotypes were estimated (Table 1). Haplotypes determined to be too rare to estimate their specific regression coefficients were pooled into a separate group with a frequency of 0.07 (results not presented).

**Table 1:** Results from haplotype analysis for the score of back and croup

Haplotypes (SNPs numbers^a^)										Coef	Freq	***p***-value	Sim. ***p***-value
1	2	3	4	5	6	7	8^a^	9	10				
G	T	C	A	T	A	T	A	A	T	−0.300	0.383	**< 0.001**	**< 0.001**
G	T	C	A	T	A	T	A	G	C	0.090	0.021	0.657	0.718
G	T	C	A	G	G	G	A	A	T	0.119	0.027	0.518	0.889
G	C	T	C	T	A	G	A	A	T	0.090	0.025	0.626	0.963
A	C	T	C	G	G	G	G	G	C	0.300	0.474	**< 0.001**	**< 0.001**

Sim. *p*-value = *p*-value adjusted by using 100,000 permutations

Significant results in bold

*Coef*. coefficient, estimated effect of the haplotype on the score of back and croup from the glm model in the haplotype analysis

*Freq*. frequencies

^a^SNP numbers in bp position order with top SNP as number 8 with reference allele A and alternate allele G

#### Phenotype association of the haplotypes with a significant effect on the score of back and croup

The t-test analyses revealed that several traits in addition to back and croup significantly differed in mean scores between horses with the favourable and unfavorable haplotype. The two haplotype groups differed significantly in mean scores (*p*-value ≤0.05) for the gait traits tölt and pace (Table 2). The two haplotype groups also differed significantly in means for the zoometric measurements of depth at breast, width of hips and thigh bones, and length of the forelimbs. In addition to this, there were significant differences between the two haplotype groups for the sub-traits backline and the croup type.

**Table 2:** Significant results from *t*-test comparing phenotypes in horses with different haplotypes

Trait	Favorable haplotype		Unfavorable haplotype				
	N	Mean	N	Mean	***t***-value	df	***p***-value
Back and croup	34	8.29	28	7.71	4.05	58.08	< 0.001
Tölt^a^	33	8.41	27	7.96	2.52	45.79	0.015
Pace^a^	33	7.18	27	6.09	2.99	50.24	0.004
Slow tölt^a^	33	8.14	26	7.73	2.14	45.19	0.038
Depth at breast (M4)^b^	33	63.2	28	64.6	−3.52	56.22	0.001
Width of the hips (M7)^b^	23	47.0	20	48.1	−2.21	37.54	0.033
Width between thigh bones (M8)^b^	23	43.0	20	44.2	−2.23	38.86	0.031
Length of forelimbs (M1-2xM4)^b^	33	15.2	28	12.1	3.22	40.81	0.003
Backline^c^	34	1.79	28	2.25	−2.69	58.91	0.009
Croup type^c^	34	1.85	28	2.18	−2.31	53.23	0.025

*N* Number of horses

^a^Subjectively assessed traits (scale 5–10)

^b^Zoometric measurements (cm)

^c^Subjectively assessed sub-traits (scale 1–3)

### Allele frequency of top SNP and *DMRT3* in different breeds

Comparing allele frequencies of the top SNP identified from GWA analysis between different breeds revealed a higher frequency of the alternate allele (the favorable allele) in the Icelandic breed compared with all other investigated breeds (Table 3).

**Table 3:** Allele frequency of top SNP for back and croup and *DMRT3*

Breed	Top SNP			***DMRT3***		
	N	AF alt	Source	N	AF alt	Source
Icelandic horses included in present study^a^	177	0.50	Array genotyping	177	0.94	Array genotyping
Icelandic horses unassessed^b^	49	0.51	SNP genotyping	49	0.90	SNP genotyping
**Other gaited breeds**						
Rocky-Mountain	36	0.33	SNP genotyping	27	1	SNP genotyping
Colombian paso horses Colombian trocha	37	0.24	Array genotyping	37	0.0	SNP genotyping
Colombian trot and gallop	11	0.23	Array genotyping	11	0.0	SNP genotyping
Colombian paso fino	38	0.29	SNP genotyping	28	1	[28]
**Partly gaited breeds**						
American Curly	27	0.32	SNP genotyping	101	0.70	[29]
American Saddlebred	42	0.29	SNP genotyping	89	0.28	[30]
Morgan	30	0.44	SNP genotyping	59	0.14	[29]
**Non- gaited breeds**						
Exmoor	279	0.01	[31]	27	0.0	[31]
Connemara Pony	40	0.05	[32]	35	0.0	[30]
Swedish Warmblood	379	0.26	[33]	64	0.0	[30, 34]
Thoroughbred racehorses	370	0.14	[35]	55	0.0	[30, 34]
Persian-Arabian horses	101	0.32	[36]	69	0.0	[30]
North-Swedish draught	25	0.38	[37]	34	0.0	[30, 34]
**Harness racing breeds**						
Coldblooded trotters	565	0.13	[38]	306	0.45	[30]
Standardbred	40	0.29	SNP genotyping	270	0.97	[30, 34]

*N* number of horses included in dataset

*Top SNP* the top SNP identified from the GWA analysis for back and croup

*AF alt* frequency of alternate allele

*DMRT3 AF alt* allele frequency of the alternate allele A in the DMRT3 gene known as the “Gait Keeper” mutation

^a^The 177 Icelandic horses included in the present study

^b^Icelandic horses used for riding but that had not attended breeding field test

### Functional annotation of genes in the region associated with the score of back and croup

The detected QTL ECA22: 45347522–45,662,708 harbors the genes *Chromosome 22 C20orf85 homolog* (*C22H20orf85*), *Ankyrin repeat domain 60* (*ANKRD60*) and *LOC100056167* described as *serine/threonine-protein phosphatase 4 regulatory subunit 1*. The SNP on ECA12 (position 26,756,656–26,756,656) was located close to the gene *solute carrier family 22 member 8* (*SLC22A8*). None of the significant SNPs (on ECA12 and 22) overlapped any known QTL for conformation in horses [39].

## Discussion

Conformation of the back and croup plays an important role for riding ability, gait ability, welfare, and longevity of the horse [1, 3, 13, 40]. The present study was performed to identify genomic regions associated with conformation of the back and croup in Icelandic horses and investigate their effects on riding ability. A novel QTL was detected on ECA22 with candidate genes associated with scoliosis and anthropometric traits in humans [41, 42]. Our results show that this QTL is of importance not only for conformation of back and croup, but also for riding ability traits, especially lateral gait quality, in Icelandic horses.

### Possible links between scoliosis, motor laterality and lateral gaits

The detected QTL for the trait back and croup harbors the genes *C22H20orf85* and *ANKRD60,* both of which are potentially linked to adolescent idiopathic scoliosis (AIS) in humans [41]. Scoliosis is defined as a lateral curvature of the spine and it is the most common vertebral disorder in children and adolescents [43]. In humans, scoliosis can be caused by muscular dystrophy or cerebral palsy, but the cause is usually unknown and therefore referred to as idiopathic [43]. AIS in humans has been shown to result in a generalized skeletal muscle weakness, respiratory impairment and exercise limitation [44]. Studies on scoliosis in humans have also shown correlation between handedness and truncal asymmetry [45–49] and that molecular basis of handedness are more likely formed by spinal gene expression asymmetries rather than in the motor cortex [50]. Symptoms of scoliosis in horses has been described as an S-shaped bend of the caudal thoracic vertebral column, resulting in restricted movements of the hind limbs and inflexibility of the back [51]. Another report described symptoms as a lateral deviation of the head and cervical and cranial thoracic vertebral column to one side, and associated rotation of the thoracic vertebrae. These deviations result in difficulties for a horse to walk in a straight line [52]. However, severe thoracic vertebral malformations in horses are infrequent, and mild to moderate forms of scoliosis may go undetected as the strong dorsal spinal musculature can mask subtle deviations of the vertebral column [52]. Scores for conformation of back and croup in horses involve both muscular and skeletal assessments, which may indicate that the back and croup phenotype shares some features with mild forms of scoliosis. It is well known that horses commonly demonstrate motor laterality (handedness) [53–55] and some even have difficulties walking in a straight line at the beginning of training. The latter often need more time in training to improve their balance and straightness.

In general, disorders of the back appear to be relatively common in horses and lead to pain and decreased performance [51]. However, to our knowledge, there are no studies reporting the prevalence of back problems or scoliosis in Icelandic horses, and it is generally hard to diagnose back pain in horses. The effect of the QTL is more likely related to functional advantage or disadvantage for movements and strength of the back and croup in horses rather than the result of more severe dysfunctions and pain. This is supported by the relatively high frequency of the unfavorable haplotype among the Icelandic horses in the present study.

### Top SNP allele frequency in other breeds

Icelandic horses had a higher frequency of the alternate allele (the favorable allele) of the top SNP for back and croup compared with all other investigated breeds, including the other gaited and partly gaited breeds. In addition, the Icelandic horses with the favorable haplotype had on average higher scores for the lateral gaits tölt and pace. Therefore, it is likely that the quality of the lateral gaits rather than the ability to perform the gaits is affected by the QTL. Almost all Icelandic horses carry at least one copy of the mutant allele A in the *DMRT3* gene known as the “Gait Keeper” mutation [30, 34]. This mutation is known to affect the pattern of locomotion in horses and the ability to perform lateral gaits [34]. The Icelandic horses in the present study had a high frequency of the *DMRT3* “Gait Keeper” mutation (0.94), 157 of the 177 horses were homozygous AA. The *DMRT3* genotype was taken into account in the phenotype association analysis. Pace scores in horses with the CA genotype were considered as a missing value. Despite this, the Icelandic horses with the favorable haplotype had higher scores for pace. This further supports our hypothesis that the detected QTL affects the quality and not the ability of lateral gaits. The genotyped gaited breed Rocky-Mountain Horse is known to be fixed for the *DMRT3* “Gait Keeper” mutation [30]. The other genotyped gaited breeds American Curly, American Saddlebred and Morgan horses have a moderate high frequency of the *DMRT3* “Gait Keeper” mutation [30, 34, 56]. These breeds are considered as partly gaited as not all horses within the breed perform ambling gaits. Trotters are also known to perform lateral gaits, and the reported frequency of the *DMRT3* mutation is high in Standardbreds (0.97–1.00) [30, 34] and relatively high in Coldblooded trotters (0.45) [30]. All of these gaited and partly gaited breeds had a higher frequency of the reference allele than the alternate allele for the top SNP of back and croup. The genotyped Colombian paso horses (CPH) included a group of horses that perform trocha and one group that only perform trot and gallop. The trocha gait is defined as a four-beat gait that includes a lateral step but it is diagonally coupled and therefore not considered a lateral gait [28, 57]. The allele frequency of the top SNP did not differ between these two groups. A group of CPH that perform the lateral gait paso fino was also genotyped. However, like all the other genotyped breeds, this group had a lower frequency of the alternate allele of the top SNP for back and croup compared to the Icelandic horses. None of the other genotyped breeds in this study segregates for the *DMRT3* mutation [30, 34], nor do they perform lateral gaits.

The 49 unassessed Icelandic horses had a similar allele frequency of the top SNP for back and croup as well as for the *DMRT3* mutation as the 177 assessed Icelandic horses included in the present study. The unassessed group included riding school horses and horses used for hobby riding. It could be argued that balance and straightness is even more essential for the training of Icelandic horses as they carry relatively heavy (adult) riders, relative to their size, in lateral gaits such as tölt and pace with strong focus on the gait quality. In addition, the Icelandic horses with the favorable haplotype had higher average scores for the lateral gaits tölt and pace, which are highly valued traits in the breed. It is likely that there has been selection for the alternate allele of the top SNP in Icelandic horses.

### Genes within the QTL associated with musculoskeletal traits

The gene *ANKRD60* is associated with body height in humans [42] and a recent study in American Miniature Horses reported a QTL for withers height close to another Ankyrin Repeat Domain gene *ANKRD1* [58]. The QTL region on ECA22 harbors the gene *LOC100056167* that is not well annotated in horses. The gene is described as serine/threonine-protein phosphatase 4 regulatory subunit 1 and appears to blast with the pseudogene *PPP4R1L* in humans with 84.17% identity [59]. The pseudogene *PPP4R1L* is transcribed in humans and *LOC100056167* has exons. *PPP4R1L* has a potential effect on bone mineral density as it has a protein phosphatase regulator activity [60]. *PPP4R1L* is regulated by an enhancer (Genehancer ID GH20J058887) with potential implications on body height and BMI-adjusted waist circumference in humans [61, 62]. Therefore, it is possible that the detected QTL effects both the muscular and skeletal system.

The horses with the favorable haplotype in the present study had longer forelimbs than those with the unfavorable haplotype. This may be explained, at least to some extent, by the effects of the genes *ANKRD60* and *LOC100056167*. According to a previous study, high-class Icelandic horses are distinguished from low-class horses by an uphill conformation [3]. High-class horses have higher withers and higher set neck and back, compared to height at croup and tuber coxae [3]. Uphill conformation is believed to facilitate ease of collection and lightness in the front part, features that are taken into account when gait quality is subjectively assessed at breeding field tests [12]. Stride length is associated with limb length in horses and other species [63–65] and stride length is also taken into account when assessing the gait quality at breeding field tests [12]. Consequently, stride length and uphill conformation are important factors for higher gait quality scores, both of which may be connected to longer forelimbs. This further supports the results from this study as the horses with the favorable haplotype had both longer forelimbs and higher scores for tölt and pace. In line with this, the horses with the unfavorable haplotype also had a deeper breast and more negative standardized marks for the sub-trait backline compared with the ones with the favorable haplotype. This indicates that a downhill conformation is more common in horses with the unfavorable haplotype. It is possible that a downhill inclination creates an imbalance between the front and back of the horse, causing difficulties for the horse to stretch the hind legs forward, thus losing the ability for self-carriage and collection. This may also result in a shorter stride length, causing lower scores for tölt and pace.

Length and form of the croup are also known to discriminate between high-class and low-class Icelandic horses [3]. In the present study, horses with the favorable haplotype had more positive standardized marks for the sub-trait croup type. This trait is defined as how evenly the croup is shaped and suggests that the haplotype does not influence the length or inclination of the croup, but only the shape of it. The difference between the two haplotype groups for the width of hips (M7) and width between the thighbones (M8) suggest that horses with the favorable haplotype may have a slimmer framed croup than horses with the unfavorable haplotype.

### Complexity of the phenotype

Until around year 2010, a soft, lower backline was considered to be favorable for the assessment of back and croup of Icelandic horses, as a low position of the back was assumed desirable for tölt [12]. A study in American Saddlebred horses detected a region on ECA20 associated with extreme lordosis (swayback) [66]. However, in the present study no significant association with back and croup was detected on ECA20. Horses with the haplotype associated with lower score of back and croup were more inclined to have a forward sloping and/or swayback backline.

The back and croup is a complex trait, with muscular as well as skeletal features of both the back and the croup subjectively assessed and scored together as a single trait. Our results show that the novel detected QTL associated with back and croup conformation influences various riding ability and conformation traits. It should be noted that the complex conformation and riding ability traits are likely to be influenced by many different genes as well as environmental factors such as feeding and training. Therefore, further studies are needed to determine the effects of this newly discovered QTL.

## Conclusions

This study provides valuable information about the genetics of conformation of the back and croup in Icelandic horses. A novel QTL for the trait back and croup was detected on ECA22: 45347522–45,662,708. The QTL is associated with the back inclination, the form of the croup, and length of limbs as well as the quality of the lateral gaits pace and tölt. These findings could result in the offering of a genetic test to aid in the selection of breeding horses, thus they are of major interest for horse breeders. The genomic region harbors genes associated with scoliosis and anthropometric traits in humans. The findings could serve as a platform to study any potential link between scoliosis and motor laterality in horses and other species. Further analyses are needed to fully understand the biological function of this genomic region on the conformation of back and croup and its influence on gait quality.

## Methods

### Animals

In total, 177 Icelandic horses (77 males and 100 females) born between 1993 and 2014 were included in the study. Hair samples were collected at breeding field tests and by visiting trainers and breeders in Iceland and Sweden. A few samples were also sent in by horse owners after personal contact and posting on social media. Only privately owned horses participated in the study and the horses were not specifically selected based on conformation of back and croup. Pedigree data were obtained from the international Icelandic horse database Worldfengur [67]. Maximum relatedness between horses was limited to half-siblings.

### Phenotyping

Phenotype data were obtained from the international Icelandic horse database Worldfengur [67]. The phenotype used for the genome-wide association (GWA) analysis consisted of the subjectively assessed score for back and croup recorded at breeding field tests between 1999 and 2018. Additional conformation and riding ability traits assessed at breeding field tests were used to investigate the effects of genomic regions detected from GWA analysis. Of the 177 horses had 115 attended more than one breeding field test. For these horses, information from the latest assessment was used. The majority of horses were assessed in year 2018 (*n* = 89). The horses were assessed in Iceland (*n* = 81), Sweden (*n* = 87), Germany (*n* = 3), Denmark (*n* = 2) and Norway (*n* = 4). Icelandic horses can attend breeding field test from when they are 4 years old. The age of assessment was on average 6.7 years and ranged from 4 to 15 years. In our sample, 173 horses were assessed for both conformation and riding ability traits, and 4 horses were only assessed for conformation traits as the ridden test is optional. Pace scores for horses with the CA genotype for the DMRT3 gene (*n* = 20) were treated as missing values.

#### Back and croup

Back and croup, along with other conformation and riding ability traits assessed at breeding field tests, were subjectively scored on a scale from 5 to 10 with 0.5 intervals, where a score of 5 was only given if a trait was not presented. Assessment of the trait back and croup comprises several aspects of the conformation of the back, croup and loins. The slope and shape of the backline, which is defined as the line from the base of withers to the lumbosacral joint, were assessed. Length and slope of the croup were also assessed, as well as the width and muscularity of the back, the length and width of the loins and the form and muscularity of the croup [12]. A high score for back and croup represents a strong, well-balanced backline and a well-muscled wide back. The croup should be long, evenly formed, well-muscled and adequately sloping. A low score is associated with a swayback, stiff or forward sloping backline, a too short or too long and/or unevenly formed croup and poorly muscled back and croup [12]. When the judging panel has reached a consensus on a score for back and croup according to the judging scale, they have the possibility to use standardized marks to describe the most prominent positive and/or negative attributes of the trait.

**Fig. 2:**
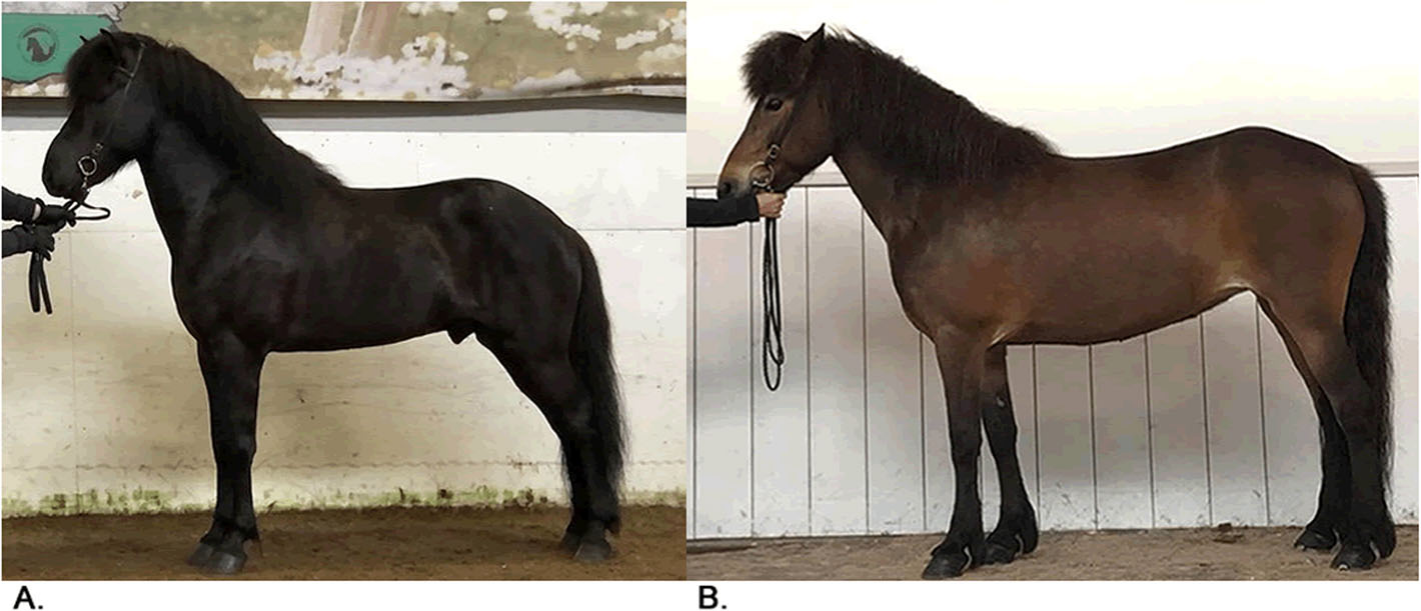
Examples of Icelandic horses representing high and low score of back and croup. **a.** Icelandic horse that represents high score of back and croup. The backline is well-balanced and the back is wide and well-muscled. (Photo: Hrefna María Ómarsdóttir). **b.** Icelandic horse that represents a low score of back and croup with a forward sloping backline and a less muscled croup. (Photo: The Swedish Icelandic Horse Association, SIF). Images used in the figure are privately owned and thus were not taken from previously published sources requiring written permission for use

**Fig. 3:**
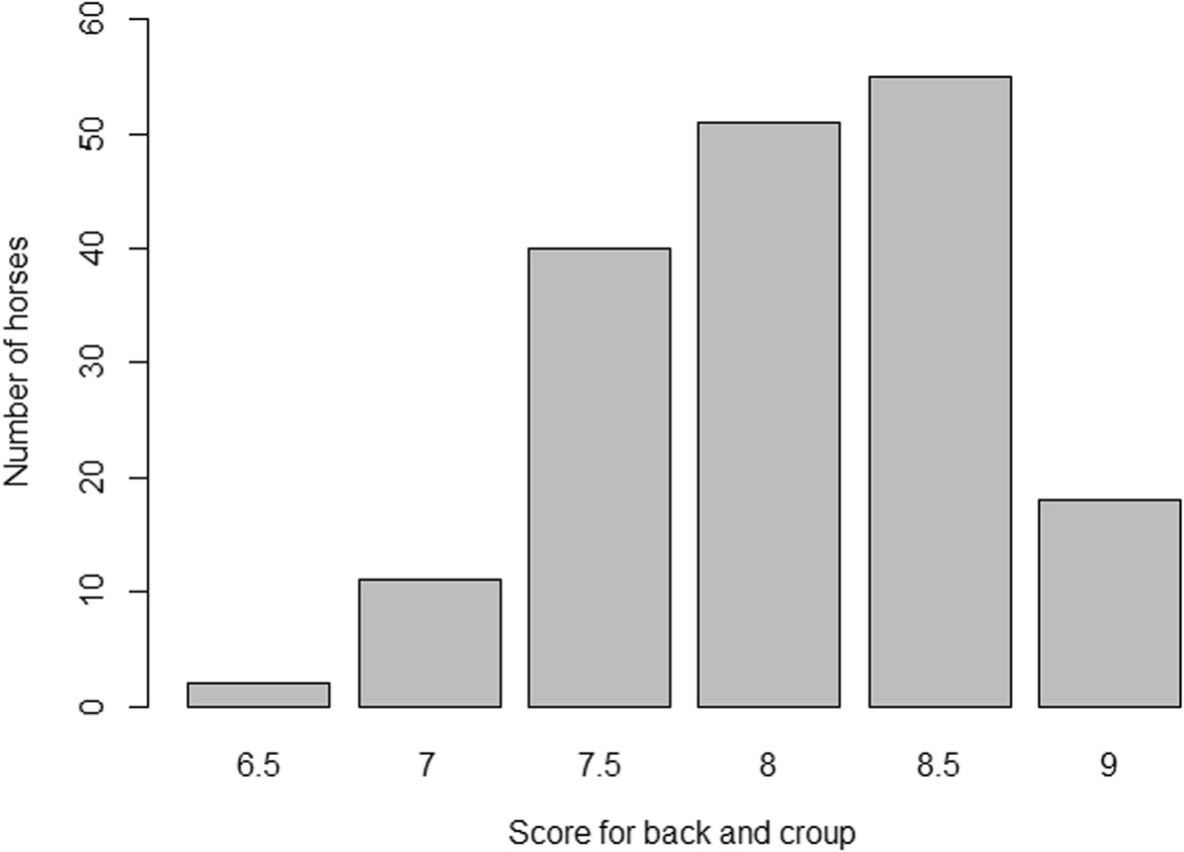
Distribution of scores for back and croup in the 177 horses. (Picture generated from the data analysis in R)

Pictures with examples of horses representing high and low score for back and croup are presented in Fig. 2. The 177 horses in the study had a score of back and croup that ranged from 6.5 to 9.0 with a mean value of 8.1 (SD 0.56) (Fig. 3). The distribution of the scores for back and croup was slightly negatively skewed (coefficient of skewness − 0.36). Transformation of the raw data to increase normality was tested but was found to not affect the results. Moreover, the residuals from the linear models were normally distributed (results not presented).

#### Sub-traits based on standardized marks for back and croup

For the purpose of more detailed analysis of the score for back and croup, the standardized marks used to describe prominent positive and negative attributes of the trait were defined as two different sub-traits; backline and croup type. These sub-traits were analysed on a linear scale ranging from 1 to 3, where 1 represented a positive mark, 3 represented a negative mark and 2 represented no mark and was interpreted as an intermediate description of the trait (not positive or negative). A positive mark for the sub-trait backline was given for good backline (well-balanced backline) and the options for negative marks were forward sloping back, straight back, sway back and/or stiff loins. For the sub-trait croup type, a positive mark was given for evenly formed croup and the options for negative marks were rounded croup, narrowing croup, roof-shaped croup and/or coarse croup.

#### Additional trait assessment scores from breeding field tests

Besides the conformation trait back and croup, scores for the gait traits tölt, slow tölt, trot, pace, gallop, canter and walk and the trait form under rider were included in this study. Features of each gait such as beat, suppleness, stride length, leg-action, speed capacity, collection and lightness were taken into account when assessing the gaits [12]. Scores of all these traits were included to investigate the effects of the detected regions from GWA analysis on the trait back and croup.

#### Zoometric traits measured at breeding field tests

**Fig. 4:**
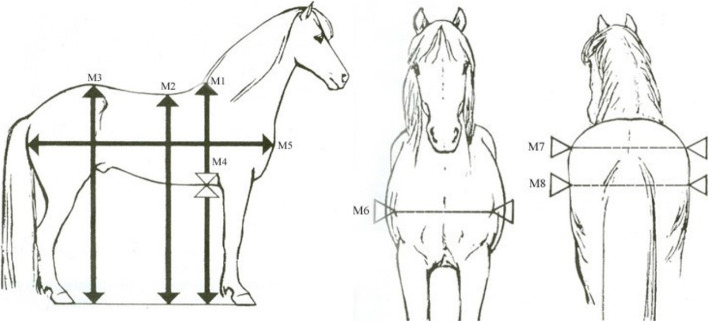
Zoometric measurements recorded at standardized breeding field tests for Icelandic horses [68]. Original images created by Pétur Behrens. Images used in the figure are privately owned and thus were not taken from previously published sources requiring written permission for use

Zoometric measurements are traditionally recorded at breeding field tests to corroborate the subjective conformation assessments [12]. All these measurements were included to investigate the effects of the detected genomic regions from GWA analysis for the trait back and croup. The measurements consisted of height at withers (M1), height at lowest point of back (M2), height at croup (M3), depth of breast (M4), length of body from the point of shoulder to tuber ischii (M5), width of chest between the points of the shoulders (M6), width of the hips between the tuber coxae (M7) and width of the hips between the hip joints (M8) (Fig. 4). Length of forelimbs is traditionally assessed from calculation of the difference between height at withers and depth at breast times two (M1-2xM4), as it gives better comparison of the leg length to consider the variation in breast depth between different horses. Other calculated measurements used for conformation assessments were difference between height at withers and height at back (M1-M2), difference between height at withers and height at croup (M1-M3), difference between height at croup and height at back (M3-M2), difference between length of the horse and height at withers (M5-M1), difference between length of the horse and height at croup (M5-M3) and difference between width of hips and width between thigh bones (M7-M8).

### DNA isolation

DNA was extracted from hair roots using a standard procedure of hair preparation. One hundred eighty-six microlitre of 5% Chelex® 100 Resin (Bio-Rad Laboratories, Hercules, CA) and 14 μL of proteinase K (20 mg/mL; Merck KgaA, Darmstadt, Germany) were added to each sample. This mix was incubated at 56 °C for 2 h at 600 rpm and proteinase K was inactivated for 10 min at 95 °C.

### Genotyping and quality control

The 177 Icelandic horses were genotyped on the 670 K+ Axiom Equine Genotyping Array. Quality control (QC) was performed with the package GenABEL [69] in R [70] to remove poorly genotyped and noisy data based on the following thresholds: missing genotypes per single nucleotide polymorphism (SNP) (> 0.10), missing SNPs per sample (> 0.10), minor allele frequency (MAF) (< 0.05) and Hardy-Weinberg equilibrium (*p*-value 1e-^10^).

### Genome-Wide Association Study (GWAS)

**Fig. 5:**
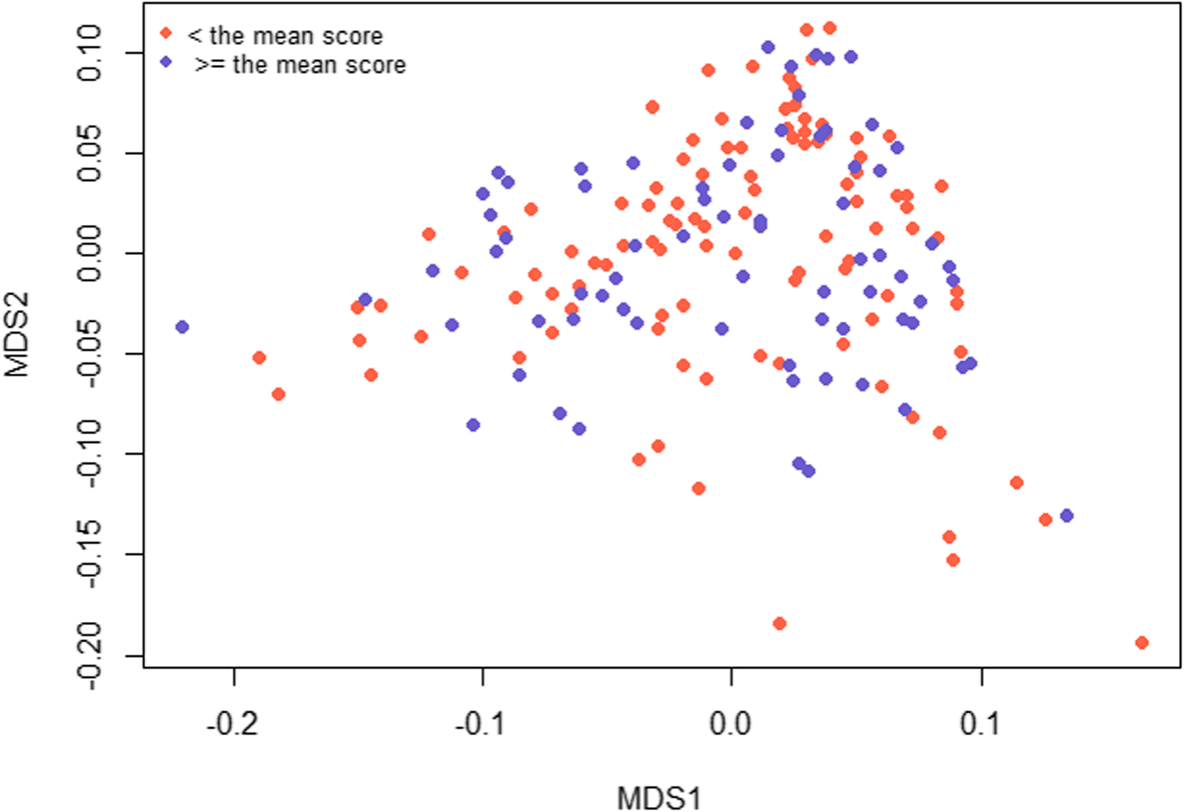
MDS plot for the score back and croup. Visualization of population stratification across the 177 Icelandic horses that passed the QC for the score back and croup. Red represents horses that had a score lower than the mean 8.1 and blue represents horses that had a score higher or equal to 8.1

GWA analyses were performed using the package GenABEL [69] in R [70]. Possible fixed effects were tested in a linear model using anova as a post hoc test. The tested fixed effects were sex (male or female), age at assessment in age classes (4, 5, 6 or ≥ 7 years old), age at assessment in years as a linear regression, country of assessment in two classes (Iceland or Sweden/other countries) and year of assessment in five classes (< 2010, 2010–2015, 2016, 2017 or 2018). The division of year of assessment classes was based on change in how the back and croup phenotype was to be assessed, and number of horses in the data from different years. The DMRT3 genotype was also tested as an effect. None of these fixed effects were found to be significant (*p* ≤ 0.05) for the trait back and croup and were thus not included in the GWA models. To investigate potential stratification, a multidimensional scaling (MDS) plot was constructed based on a genomic relationship matrix using the GenABEL package and ibs() function [69]. No outliers were apparent on the MDS plot and no stratification of horses with low and high score of back and croup was detected. A visualization of the genomic-kinship matrix using MDS is shown in Fig. 5.

The genomic-kinship matrix together with the phenotype of back and croup were passed to the polygenic_hglm function using family gaussian in GenABEL [69, 71]. To account for any population stratification, the GWA analysis was performed using a mixed model-structured association approach with the mmscore function in GenABEL [69]. Genome-wide significance was determined by Bonferroni correction and a suggestive genome-wide significance threshold was set at 1.0 × 10^− 5^ [72, 73]. QQ and linkage disequilibrium (LD) manhattan plots were performed using the package cgmisc 2.0 [74].

### Haplotype analysis

Haplotype analysis was performed with the haplo.stats package in R [70]. A linkage disequilibrium plot was constructed and the ten significant SNPs in LD (r^2^ ≥ 0.8) were used in the function haplo.em to estimate haplotypes. The haplotype effect on the score of back and croup was estimated by a gereralized linear model (glm) with the function haplo.glm.. The most frequent haplotype was used as a reference and only haplotypes with frequencies greater than 0.02 were included. A simulated *p*-value was estimated by using 100,000 permutations considering an additive effect.

#### Phenotype association of significant haplotypes

Phenotype association of the horses homozygous for the haplotypes that had a significant effect on the conformation of back and croup was performed using a two-tailed Student’s t-test in R [70]. Significance level was set at p-value ≤0.05. Traits tested were all the zoometric traits, the subjectively scored riding ability traits and the subjectively assessed sub-traits.

### Genotyping of the top SNP and *DMRT3* in other gaited and partly gaited breeds

Horses of other gaited breeds (Rocky-Mountain: 36 horses, Colombian paso fino horses: 38 horses) and partly gaited breeds (American Curly: 27 horses, American Saddlebred: 42 horses, Morgan: 30 horses and Standardbred: 40 horses) were genotyped for the top SNP using StepOnePlus Real-Time PCR System (Life Technologies) with a custom TaqMan SNP genotyping assay (Applied Biosystems). A group of 49 Icelandic horses used for riding but that had not attended breeding field test was also genotyped. The sequence of the primers and probes was designed as follows: forward primer: 5′-GGAAGTTTCTAAACATTTTTGAAGGCTTTT-3′; reverse primer: GGAGGGAAGTCAATTGACAAACG; mutant probe (FAM): 5′-CCTCCACGGCATCA-3′; reference probe (VIC): 5′-TCCCTCCACAGCATCA-3′. The reaction volume of 15 μl contained: 1.5 μl DNA, 0.38 μl Genotyping Assay 40X, 7.50 μl Genotyping Master Mix 2X, and 5.62 μl deionized water. The thermal cycle included 95 °C for 10 min, 40 cycles of 95 °C for 15 s, and 60 °C for 1 min.

SNP genotyping of the *DMRT3*_*Ser301STOP* marker known as the “Gait Keeper” mutation was performed using custom designed TaqMan SNP Genotyping Assays (Applied Biosystem) as described previously [30, 34].

### Functional annotation

The bioinformatics database NCBI was used to screen for candidate genes based on the EquCab3.0 reference genome and annotation release 103 [75] and HorseQTLdb release 41 to search for known quantitative trait loci (QTLs) for conformation in horses [39]. Functional annotation of possible candidate genes was performed using the GeneCards database [76]. All positions refer to the EquCab3.0 reference genome.

## Supplementary Information

**Additional file 1.**

## Data Availability

The datasets generated and analyzed during the current study are not publicly available since the study was performed in collaboration with the Icelandic horse breeding industry and has a commercial value for them. However, data is available from the corresponding author on reasonable request and with permission of the Icelandic horse association.

## Abbreviations

*ADAMTS17*: *ADAM Metallopeptidase With Thrombospondin Type 1 Motif 17*
AF: Allele frequency
AIS: Adolescent idiopathic scoliosis
*ANKRD1*: *Ankyrin Repeat Domain 1*
*ANKRD60*: *Ankyrin repeat domain 60*
BLAST: Basic Local Alignment Search Tool
*C22H20orf85*: *Chromosome 22 C20orf85 homolog*
CPH: Colombian paso horses
*DMRT3*: *Doublesex And Mab-3 Related Transcription Factor 3*
ECA: *Equus caballus* chromosome
*GH1*: *Growth Hormone 1*
glm: Generalized linear model
GWA: Genome-wide association
*HMGA2*: *High Mobility Group AT-Hook 2*
*LASP1*: *LIM And SH3 Protein 1*
*LCORL*: *Ligand Dependent Nuclear Receptor Corepressor Like*
LD: Linkage disequilibrium
MAF: Minor allele frequency
MDS: Multidimensional scaling
*NCAPG*: *Non-SMC Condensin I Complex Subunit G*
*OSTN*: *Osteocrin*
*PPP4R1L*: *Protein Phosphatase 4 Regulatory Subunit 1 Like*
QC: Quality control
QTL: Quantitative trait loci
SD: Standard deviation
SIF: The Swedish Icelandic Horse Association
*SLC22A8*: *Solute carrier family 22 member 8*
SNP: Single nucleotide polymorphism
VR: The Swedish Research Council
*ZFAT*: *Zinc Finger And AT-Hook Domain Containing*
